# Prescribing 6-weeks of running training using parameters from a self-paced maximal oxygen uptake protocol

**DOI:** 10.1007/s00421-018-3814-2

**Published:** 2018-02-12

**Authors:** James S. Hogg, James G. Hopker, Sarah L. Coakley, Alexis R. Mauger

**Affiliations:** 1 Endurance Research Group, Faculty of Science, School of Sport and Exercise Sciences, University of Kent, Chatham Maritime, Chantam, ME4 4AG Kent UK; 2 Department of Sport and Exercise Sciences, University of Chichester, Chichester, UK

**Keywords:** Recreational runners, Running performance, Critical speed, Endurance training, Lactate threshold

## Abstract

**Purpose:**

The self-paced maximal oxygen uptake test (SPV) may offer effective training prescription metrics for athletes. This study aimed to examine whether SPV-derived data could be used for training prescription.

**Methods:**

Twenty-four recreationally active male and female runners were randomly assigned between two training groups: (1) Standardised (STND) and (2) Self-Paced (S-P). Participants completed 4 running sessions a week using a global positioning system-enabled (GPS) watch: 2 × interval sessions; 1 × recovery run; and 1 × tempo run. STND had training prescribed via graded exercise test (GXT) data, whereas S-P had training prescribed via SPV data. In STND, intervals were prescribed as 6 × 60% of the time that velocity at $\dot {V}{{\text{O}}_{{\text{2max}}}}$ ($_{{\text{v}}}\dot {V}{{\text{O}}_{{\text{2max}}}}$) could be maintained (*T*_max_). In S-P, intervals were prescribed as 7 × 120 s at the mean velocity of rating of perceived exertion 20 (_v_RPE20). Both groups used 1:2 work:recovery ratio. Maximal oxygen uptake ($\dot {V}{{\text{O}}_{{\text{2max}}}}$), $_{{\text{v}}}\dot {V}{{\text{O}}_{{\text{2max}}}}$, *T*_max, v_RPE20, critical speed (CS), and lactate threshold (LT) were determined before and after the 6-week training.

**Results:**

STND and S-P training significantly improved $\dot {V}{{\text{O}}_{{\text{2max}}}}$ by 4 ± 8 and 6 ± 6%, CS by 7 ± 7 and 3 ± 3%; LT by 5 ± 4% and 7 ± 8%, respectively (all *P* < .05), with no differences observed between groups.

**Conclusions:**

Novel metrics obtained from the SPV can offer similar training prescription and improvement in $\dot {V}{{\text{O}}_{{\text{2max}}}}$, CS and LT compared to training derived from a traditional GXT.

## Introduction

The graded exercise test (GXT) is a globally recognised test which offers valuable information on key aerobic parameters such as maximal oxygen uptake ($\dot {V}{{\text{O}}_{{\text{2max}}}}$), and can be used to prescribe training for both elite athletes, and recreational exercisers. Recently, a novel approach to the traditional GXT has been proposed, termed the self-paced $\dot {V}{{\text{O}}_{{\text{2max}}}}$ test (SPV), which consists of 5 × 2 min stages where speed or power is freely adjusted by the participant based on rating of perceived exertion (RPE) (Mauger and Sculthorpe 2012; Borg 1982). The SPV has been applied across a wide range of exercise modalities and ergometry despite its relative infancy (Mauger and Sculthorpe 2012; Chidnok et al. 2013; Straub et al. 2014; Hogg et al. 2015; Jenkins et al. 2017b; Lim et al. 2016; Scheadler and Devor 2015).

The general consensus from published research to date suggests that the SPV provides comparable $\dot {V}{{\text{O}}_{{\text{2max}}}}$ values to the GXT (Chidnok et al. 2013; Hogg et al. 2015; Lim et al. 2016; Scheadler and Devor 2015; Straub et al. 2014; Faulkner et al. 2015; Hanson et al. 2016), however, the methodological differences and contrasting populations used may make direct comparisons between studies challenging. Higher $\dot {V}{{\text{O}}_{{\text{2max}}}}$ values have been observed within the SPV test (Mauger and Sculthorpe 2012; Jenkins et al. 2017a, b; Astorino et al. 2015; Mauger et al. 2013), although all but one of these studies were cycling-based. However, the findings regarding differences in $\dot {V}{{\text{O}}_{{\text{2max}}}}$ are less meaningful in terms of the utility of the test, with perhaps greater emphasis being placed on the practical advantages that the SPV has over the GXT. The problems associated with the GXT are well documented (Noakes 2008), such as the incremental fixed-intensity nature of the test, unknown test duration, and creating a test environment that is possibly unnatural and irrelevant for “real” sporting performance. It has, therefore, been put forward that the SPV may represent a paradigm shift in $\dot {V}{{\text{O}}_{{\text{2max}}}}$ testing (Beltz et al. 2016), with self-paced protocols offering greater ecological validity due to the self-paced and closed-loop nature, whilst also circumventing the issue of estimating the ramp-rate and starting work rate for the researcher or practitioner (Poole and Jones 2017).

The GXT offers additional metrics in addition to the measurement of $\dot {V}{{\text{O}}_{{\text{2max}}}}$, such as the velocity at $\dot {V}{{\text{O}}_{{\text{2max}}}}$ (${}_{{\text{v}}}\dot {V}{{\text{O}}_{{\text{2max}}}}$) and the time in which ${}_{{\text{v}}}\dot {V}{{\text{O}}_{{\text{2max}}}}$ can be maintained (*T*_max_). However, the identification of *T*_max_ requires an additional test which adds to the impracticality of the GXT. Nevertheless, $\dot {V}{{\text{O}}_{{\text{2max}}}}$, ${}_{{\text{v}}}\dot {V}{{\text{O}}_{{\text{2max}}}}$ and *T*_max_ have been shown to be useful and viable parameters in running training and performance (Billat and Koralsztein 1996; Esfarjani and Laursen 2007; Manoel et al. 2017; Smith et al. 2003) and can be used to prescribe training and assess training adaptation. If similar metrics for training prescription could be acquired from the SPV, in a singular test, it would demonstrate utility over and above traditional GXT assessment of $\dot {V}{{\text{O}}_{{\text{2max}}}}$, especially as the SPV is an effective test for highly trained runners (Hogg et al. 2015; Scheadler and Devor 2015), and has good test–retest reliability (Jenkins et al. 2017a). In addition, the SPV has recently been validated as a field test (Lim et al. 2016), which increases its accessibility to a variety of athletes and coaches. Therefore, the ability to prescribe training from the SPV would enhance the value and utility of the test. As such, this study aimed to investigate whether training prescribed via novel metrics derived from the SPV could result in comparable improvements in key aerobic parameters as training formulated from traditional GXT variables.

## Materials and methods

### Participants

Twenty-four recreationally active male (*n* = 16) and female runners (*n* = 8) (Mean ± SD: Age = 30 ± 9 years, body mass = 70 ± 13 kg, height = 172 ± 9 cm) volunteered to participate in this study. Sample size was estimated from power calculations (G-Power software, Franz Faul, Universitat Kiel, Germany) with mean and SD data from a similar training study (Esfarjani and Laursen 2007). The study was conducted with the approval of the Ethics Committee of the School of Sport and Exercise Sciences at the University of Kent (Approval reference: Prop01.2014-15). All participants who volunteered read and signed a form of written informed consent before participation.

### Exercise tests

Participants were randomly allocated into two groups: ‘Standardised’ (STND) and ‘Self-paced’ (S-P). All participants completed a GXT, an SPV, a sub-maximal lactate threshold (LT) test on a motorised treadmill (Saturn, HP Cosmos, Nussdorf-Traunstein, Germany), and a critical speed (CS) test as part of baseline testing on three separate occasions over a 2 weeks period. The $\dot {V}{{\text{O}}_{{\text{2max}}}}$ protocols were completed in a randomised order, 2–7 days apart and at the same time of day (± 2 h). Oxygen uptake ($\dot {V}{{\text{O}}_{\text{2}}}$) (Metalyzer 3BR2, Cortex, Lepzig, Germany) and heart rate (T31, Polar Electro Inc, New York, USA) were recorded for the duration of the testing protocol. The online gas analysis system was calibrated prior to every test in accordance with the manufacturer’s guidelines. Before each test, participants performed a warm-up of their choice on the motorised treadmill, which was kept the same for all subsequent tests. The CS test was completed on an all-weather synthetic 400 m running track using the method outlined by Galbraith (2011). Briefly, this involved three runs at distances of 3600, 2400 and 1200 m, each separated by 30 min recovery. For the lactate threshold (LT) protocol, participants completed 4 min stages on the treadmill with a capillary blood sample (Biosen C-Line, EKF Diagnostics, Barleben, Germany) taken at the end of each stage, with the velocity increasing by 1 km h^−1^ at the beginning of each stage. Starting speed was estimated based on each participant’s individual fitness level. The test was terminated once lactate threshold 1 (LT1) and lactate threshold 2 (LT2) had been obtained, defined as blood lactate readings of 2 and 4 mmol L^−1^, respectively. Before each test, participants were instructed to maintain similar eating habits, abstain from alcohol (24 h) and caffeine (8 h), and to avoid exhaustive or vigorous exercise (48 h). These conditions were verbally verified by the experimenter at each test visit. Following baseline testing all participants then undertook a 6 weeks field-based training program, consisting of two high intensity interval training sessions, one recovery run, and a tempo run per wk. Training sessions were either based on data from the SPV or GXT [depending on group allocation]. Participants completed either a GXT, or SPV mid-training [depending on group allocation] in the third week of the training programme. This test replaced one of the high intensity sessions for that week, with its sole purpose to recalibrate interval session intensity in both groups. All baseline tests were then repeated in the immediate two-weeks that followed the 6 weeks training intervention.

#### Graded exercise test (GXT)

The test commenced at a submaximal speed, gauged by the experimenter and subject, to help bring about volitional exhaustion within 8–12 min. Speed was increased by 1 km h^− 1^ every 2 min and the test was terminated when participants reached volitional exhaustion. Treadmill gradient was set to 1%. All previously described cardiorespiratory measures were recorded during this stage and participants continued until volitional exhaustion. 6–20 RPE (Borg 1982) was recorded 20 s before the end of each stage. Verbal encouragement was given throughout. ${}_{{\text{v}}}\dot {V}{{\text{O}}_{{\text{2max}}}}$ was determined as the highest velocity that could be maintained for at least 30 s (Smith et al. 2003).

#### Determination of *T*_max_

For the GXT, the time that ${}_{{\text{v}}}\dot {V}{{\text{O}}_{{\text{2max}}}}$ could be maintained (*T*_max_) was measured in a separate bout of exercise (Smith et al. 2003). After a 20 min recovery (Nolan et al. 2014) following the GXT, participants warmed up on the treadmill at 60% ${}_{{\text{v}}}\dot {V}{{\text{O}}_{{\text{2max}}}}$ for 5 min. Participants were then allowed to stretch before remounting the treadmill with the speed being ramped up over 30 s until ${}_{{\text{v}}}\dot {V}{{\text{O}}_{{\text{2max}}}}$ was reached. Participants were then asked to continue until volitional exhaustion. Heart rate and expired gas were recorded throughout this test.

#### Self-Paced $\dot {V}{{\text{O}}_{{\text{2max}}}}$ Test

The SPV was completed as previously described by Hogg and colleagues (2015). Briefly, the SPV consisted of 5 × 2 min continuous stages with RPE increments of 11, 13, 15, 17 and 20. A zonal pacing system was used where the researcher would adjust the running speed based on the participant’s positioning on the treadmill. Participants were informed about the self-pacing zones before the warm-up and then practiced moving between the zones after completing their individualised warm-up. Familiarisation of the 6–20 RPE scale and how to vary their speed according to a fixed RPE was provided via verbal explanation prior to the warm-up with specific emphasis given to considering their RPE for each given moment.

#### Determination of $\dot {V}{{\text{O}}_{{\text{2max}}}}$

Averaging of $\dot {V}{{\text{O}}_{\text{2}}}$ during GXT and SPV tests was performed over 30 s. $\dot {V}{{\text{O}}_{2\hbox{max} }}$ in the GXT and SPV was defined as the highest $\dot {V}{{\text{O}}_{\text{2}}}$ averaged for 30 s. A plateau in $\dot {V}{{\text{O}}_{\text{2}}}$ during the GXT was accepted if the change in $\dot {V}{{\text{O}}_{\text{2}}}$ during the highest 30 s average from each of the final two stages of the test were less than half of the normal stage-to-stage difference in $\dot {V}{{\text{O}}_{\text{2}}}$ during the initial linear parts of the test for each subject (Beltrami et al. 2012). As an ancillary method to verify attainment of $\dot {V}{{\text{O}}_{\text{2}}}$, secondary criteria were accepted when two of the following were attained: Heart rate (HR) within 10 bpm of age-predicted maximum; Respiratory exchange ratio (RER) ≥ 1.15 and RPE ≥ 17.

### Training programme

All participants completed two high-intensity interval sessions per week, along with a recovery run and a tempo run. This equated to four exercise sessions per week. Participants were free to schedule the sessions throughout each week but were encouraged to not complete interval sessions and tempo run on consecutive days. All sessions were completed using an assigned global positioning system (GPS) watch (310XT, Garmin International Inc, KS, USA), and training was logged in a training diary.

#### STND Group

For each interval session, participants completed 6 intervals at ${}_{{\text{v}}}\dot {V}{{\text{O}}_{2\hbox{max} }}$ with duration determined as 60% of *T*_max_ (Smith et al. 2003). A 2:1 ratio was used to determine the recovery stage duration in-between each interval. Recovery run intensity was calculated as 60% of their maximal heart rate (HR_max_) obtained from the GXT. Participants were required to run for 30 min. This session was included to help ensure participants would not be encouraged to supplement their program with additional training.

Tempo run intensity was determined from the submaximal LT test and participants were required to run at a velocity calculated as 50% between LT1 and LT2 for 30 min.

#### S-P group

For each interval session, participants completed 7 × 2 min intervals at a velocity corresponding to the mean velocity completed during the final (RPE20) stage of the SPV. A 2:1 ratio was used to determine the recovery stage duration in-between each interval. The recovery run was the same as in the STND group, but intensity was calculated as 60% of their HR_max_ obtained from the SPV.

Tempo run intensity was determined by calculating the ventilatory threshold (VT) via the V-Slope method from the $\dot {V}{{\text{O}}_2}$ and $\dot {V}{\text{C}}{{\text{O}}_2}$ data collected during the SPV (Beaver et al. 1986). The participants were then asked to run at an RPE that corresponded with the stage of the SPV in which the VT was achieved. The participants were asked to freely adjust their pacing to match the required RPE.

### Statistical analysis

Prior to statistical analysis, data were checked and confirmed to be normally distributed. A paired samples *t* test was performed to assess maximal value differences between protocols. Based on the achieved effect size, a post hoc power analysis demonstrated that the statistical power of the pre-post $\dot {V}{{\text{O}}_{{\text{2max}}}}$ comparison was 0.93. To identify training responses for both training groups (group) and GXT and SPV protocols (protocol) for before and after training (time-point) a mixed model analysis of variance (ANOVA) was used. Where no interaction effect was identified between a variable and protocol (GXT and SPV), the protocol was omitted from further analysis of training responses for that variable. Participants’ CS were calculated from the field test using a linear distance-time model. Partial eta-squared ($\eta _{p}^{2}$) was used to report effect sizes, and statistical significance was accepted when *P* < .05. All statistical tests were completed using SPSS version 24 (Chicago, IL, USA).

## Results

### SPV vs. GXT protocol data

#### Incidence of $\dot {V}{{\text{O}}_{\text{2}}}$ plateau in GXT and SPV Protocols

The average stage-to-stage increase in $\dot {V}{{\text{O}}_{\text{2}}}$ for all participants was calculated as 393 ± 21 mL min^− 1^, so that a plateau phenomenon was defined as a change in $\dot {V}{{\text{O}}_{\text{2}}}$ ≤ 197 ± 10 mL.min^− 1^ (or relative $\dot {V}{{\text{O}}_{\text{2}}}$ 2.8 mL kg^− 1^ min^− 1^), between the highest 30 s average obtained from each of the final two stages of the test for each participant. All participants achieved either a $\dot {V}{{\text{O}}_{\text{2}}}$ plateau or satisfied secondary criteria across both GXT trials before and after training. Ninety-three percent of participants satisfied secondary criteria across both SPV trials before and after training.

#### Differences in test protocols

Differences in test protocols for key variables for all participants are presented in Table 1. Pre and post-training data were combined to compare the GXT and SPV protocols. There were no significant differences in $\dot {V}{{\text{O}}_{2\hbox{max} }}$ between the GXT and SPV protocols (*P* = .578). Maximal RER (RER_max_) was significantly greater in the SPV compared to the GXT (*P* < .001). There was no interaction effect between test protocol for either HR_max_ or maximal minute ventilation (*V*_Emax_) (*P* = .212; *P* = .319, respectively). Protocol duration was significantly longer in the GXT (*P* < .001). RPE_max_ was significantly greater in the SPV (*P* < .001). There were no significant differences between the velocities associated with $\dot {V}{{\text{O}}_{2\hbox{max} }}$ and RPE20 (*P* = .130).

**Table 1:** Mean ± SD peak values for physiological and intensity variables recorded during both GXT and SPV protocols across both before and after training for all participants

Variable	Protocol	
	GXT	SPV
$\dot {V}{{\text{O}}_{{\text{2max}}}}$ (mL kg^−1^ min^−1^)	54 ± 5.8	54 ± 0.7
HR_max_ (beats min^−1^)	186 ± 12	184 ± 11
*V*_Emax_ (L min)	135.4 ± 29.4	137.2 ± 24.8
RER_max_	1.15 ± 0.02	1.21 ± 0.00*
${}_{{\text{v}}}\dot {V}{{\text{O}}_{2\hbox{max} }}$/_v_RPE20 (km h^−1)^	14.8 ± 1.3	15 ± 1.5
Mean test time (min)	11 ± 1*	10 ± 0
RPE_max_	19 ± 1	20 ± 0*

*Denotes significant difference within the group for the given variable between pre and post testing (*P* < .05)

### STND vs. S-P training data

#### Training prescription

Total prescribed training duration over the 6 weeks period for both training groups was not significantly different (*P* = .651). The STND had a prescribed total duration of 804 ± 90 min whilst the S-P had a prescribed total duration of 816 ± 0 min. There was no significant difference between the mean interval session duration for both STND and S-P (37 ± 8 vs. 38 ± 0 min, respectively) (*P* = .679).

**Table 2:** Mean ± SD maximal values for physiological and threshold variables recorded before and after training for both training groups

Variable	Training group			
	Standardised (STND)		Self-Paced (S-P)	
	Pre	Post	Pre	Post
$\dot {V}{{\text{O}}_{{\text{2max}}}}$ (mL.kg^−1^.min^−1^)	54 ± 5.0	56.3 ± 6.2*	51.7 ± 5.3	54.8 ± 5.7*
*V*_Emax_ (L/min)	130.2 ± 22.6	134.7 ± 20.4*	134.3 ± 28.7	141.5 ± 29.0*
HR_max_ (beats/min)	190 ± 13	188 ± 13	181 ± 13	182 ± 9
Critical speed (m s^− 1^)	3.47 ± 0.03	3.70 ± 0.03*	3.47 ± 0.04	3.59 ± 0.05*
LT1 (km h^− 1^)	10 ± 1.2	10.5 ± 1.2*	9.7 ± 1.5	10.5 ± 1.3*
LT2 (km h^− 1^)	11.7 ± 1.2	12.2 ± 0.8*	11.1 ± 1.8	12.1 ± 1.5*

In the STND all data is provided via the GXT and by the SPV for the S-P

*Denotes significant difference within the group for the given variable between pre and post testing (*P* < .05)

#### Responses to training

**Fig. 1:**
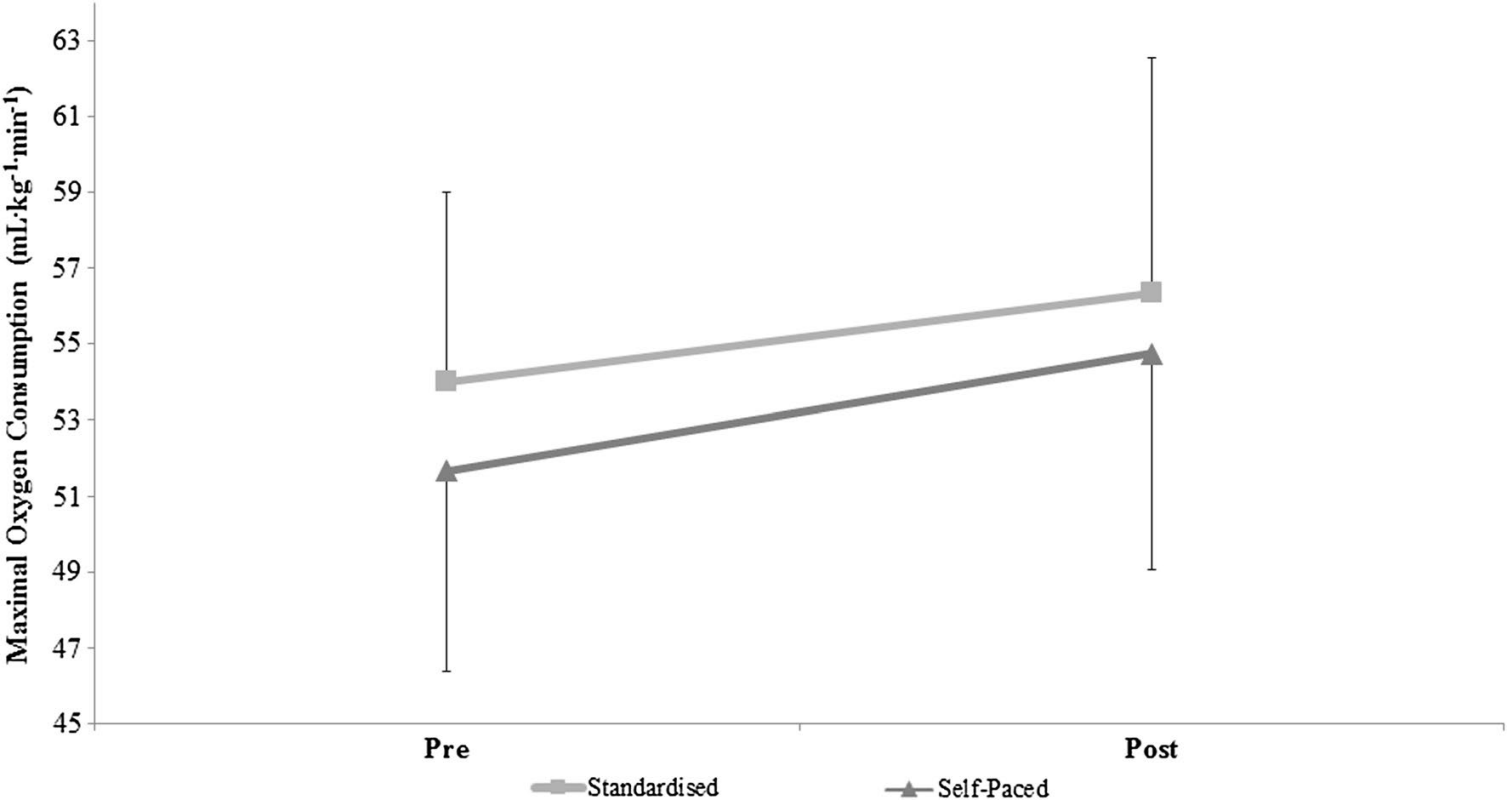
Mean ± SD Differences in VO_2max_ between the STND and S-P training groups before and after training

Group data (pre- vs. post-training) are shown in Table 2. As outlined in the methods, participants were grouped into either S-P or STND, and conducted both an SPV and GXT before and after the training intervention. There was no interaction effect for protocol duration between time-point, protocol and group (*F*_1,22_ = 0.561, *P* = .462, $\eta _{p}^{2}$ = 0.025). As shown in Fig. 1 and Table 2, there was an interaction effect between $\dot {V}{{\text{O}}_{2\hbox{max} }}$ and time-point (*F*_1,22_ = 7.461, *P* = .012, $\eta _{p}^{2}$ = 0.253), however, there was no interaction effect observed between group and time-point (*F*_1,22_ = 0.003, *P* = .954, $\eta _{p}^{2}$ = 0.0001). Whilst there was an interaction effect between *V*_Emax_ and time-point (*F*_1,22_ = 12.592, *P* = .002, $\eta _{p}^{2}$ = 0.364), there was no interaction effect between time-point and group (*F*_1,22_ = 0.001, P = .981, $\eta _{p}^{2}$ = 0.0001). There was no interaction effect for HR_max_ between time-point and group (*F*_1,22_ = 1.063, *P* = .314, $\eta _{p}^{2}$ = 0.046)

**Fig. 2:**
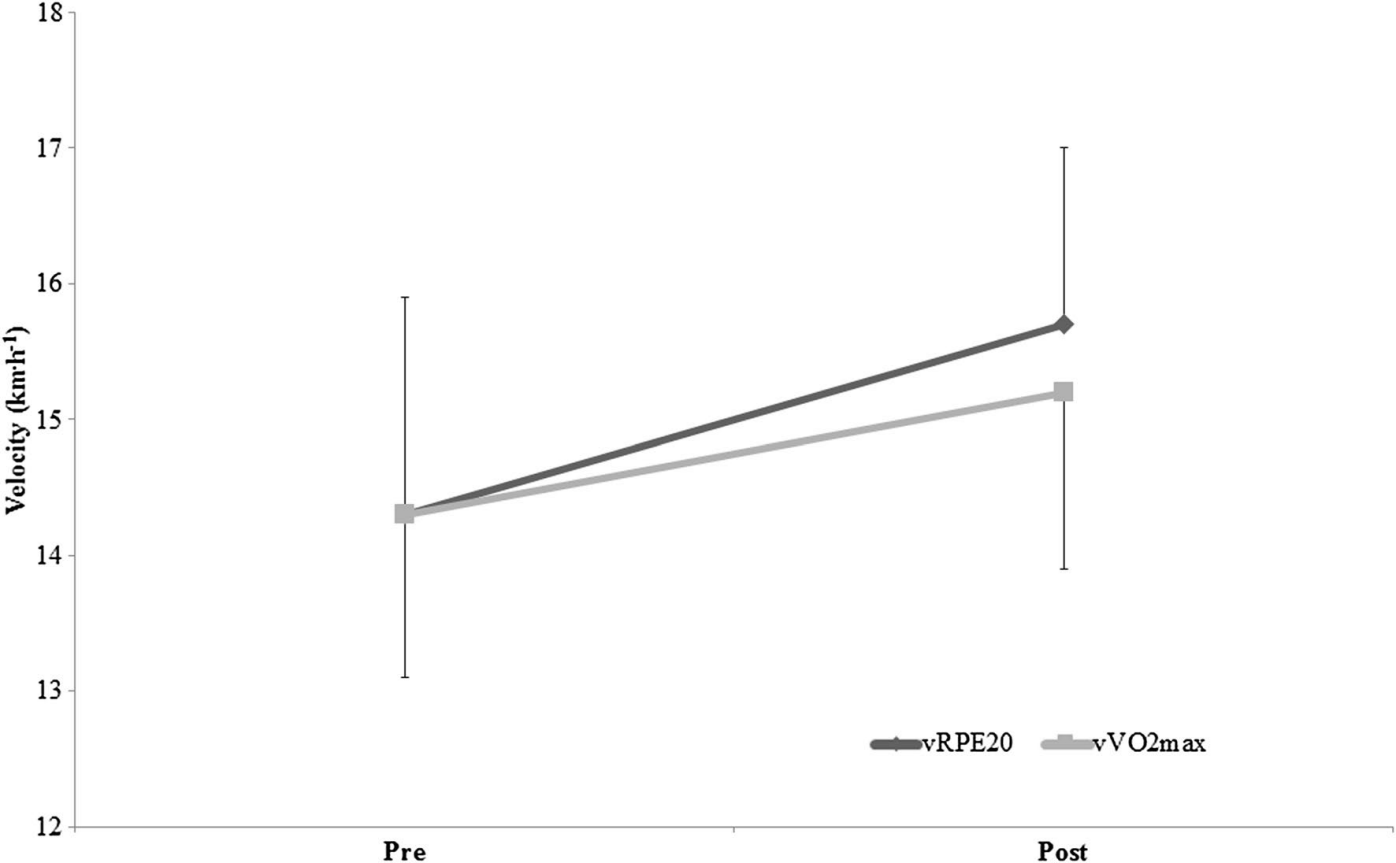
Mean ± SD Differences in the velocities ${}_{{\text{v}}}\dot {V}{{\text{O}}_{2\hbox{max} }}$ and _v_RPE20 for all participants for before and after training

There was an interaction effect between time-point and running velocity at _v_RPE20 and ${}_{{\text{v}}}\dot {V}{{\text{O}}_{2\hbox{max} }}$ *F*_1,20_ = 5.800, *P* = .026, $\eta _{p}^{2}$ = 0.225). As shown in Fig. 2, for both groups there were no differences in ${}_{{\text{v}}}\dot {V}{{\text{O}}_{2\hbox{max} }}$ and _v_RPE_20_ before training (14.3 + 1.3 vs. 14.3 + 1.7 km h^− 1^, respectively), but _v_RPE20 was greater than ${}_{{\text{v}}}\dot {V}{{\text{O}}_{2\hbox{max} }}$ after training (15.7 + 1.3 vs. 15.2 + 1.3 km h^− 1^, respectively). CS improved in both groups (*P* < .001) however there was no interaction effect between time-point and group (*F*_1,21_ = 3.006, *P* = .098, $\eta _{p}^{2}$ = 0.125). Similarly, LT1 and LT2 improved in both groups (*F*_1,21_ = 14.637, *P* < .001, $\eta _{p}^{2}$ = .411), however, there was no interaction effect between time-point and group (*F*_1,21_ = 1.227, *P* = .281, $\eta _{p}^{2}$ = .055).

## Discussion

The primary finding of this study was that following a 6 weeks period of training, recreational runners' aerobic fitness and running performance was increased by a similar magnitude, regardless of whether SPV or GXT data were used to prescribe training. Specifically, $\dot {V}{{\text{O}}_{2\hbox{max} }}$ in the STND group improved by 4%, and by 6% in the S-P group. An improvement in $\dot {V}{{\text{O}}_{2\hbox{max} }}$ in the region of ~ 3% has previously been defined as a meaningful improvement in performance (Kirkeberg et al. 2010), as opposed to day-to-day variation. Previous literature has shown improvements in $\dot {V}{{\text{O}}_{2\hbox{max} }}$ by ~ 6% when training at 106% ${}_{{\text{v}}}\dot {V}{{\text{O}}_{2\hbox{max} }}$ (Franch et al. 1998) for similar training durations. However, in the aforementioned study the starting $\dot {V}{{\text{O}}_{2\hbox{max} }}$ for the participants were significantly lower than those reported in the current study, which may suggest a greater level of trainability for $\dot {V}{{\text{O}}_{2\hbox{max} }}$ (Swain and Franklin 2002) compared with the participants in the current study. Athletes of slightly higher training status’ than those in the current study achieved little to no improvements in $\dot {V}{{\text{O}}_{2\hbox{max} }}$ over 4–6 weeks of similar intensity training (Manoel et al. 2017; Smith et al. 2003; Denadai et al. 2006), but did show significant improvements in LT and 3–10 km running performance. Similar running programmes utilising interval training have also produced improvements in CS (Esfarjani and Laursen 2007). This is supported by the findings of the current study that in both STND and S-P, CS improved by 7 and 3%, respectively. For LT1 and LT2, STND improved by 5 and 3% and S-P improved by 7 and 8%.

An important finding of this study is that the novel training parameter extracted from the SPV, ‘_v_RPE20’, is effective at prescribing running intensity for interval training. The ${}_{{\text{v}}}\dot {V}{{\text{O}}_{2\hbox{max} }}$ for the STND before and after training was 14.3 ± 0.9 vs. 15.2 ± 1.0 km h^− 1^ compared to the S-P’s _v_RPE_20_ of 14.2 ± 1.9 vs. 15.7 ± 1.9 km h^− 1^, respectively. It is likely that the _v_RPE20 may reflect a speed between ${}_{{\text{v}}}\dot {V}{{\text{O}}_{2\hbox{max} }}$ and the maximal velocity achieved in a GXT (*V*_max_). *V*_max_ has recently been shown to be as beneficial as ${}_{{\text{v}}}\dot {V}{{\text{O}}_{2\hbox{max} }}$ for exercise prescription (Manoel et al. 2017), and like _v_RPE20 is simple to calculate. Moreover, _v_RPE20 has been shown to be repeatable regardless of the pacing strategy adopted during this final stage (Hanson et al. 2017). This should be reason to encourage further investigation to assess the potential of _v_RPE20 in training prescription and its suitability as a performance parameter.

As the aim of the study was to investigate whether SPV-derived training parameters could offer similar improvements in aerobic fitness compared to GXT prescribed training, it was important that training prescription was similar between groups in both intensity and duration. To calculate interval duration for the STND, 60% *T*_max_ was used. Setting interval duration at 60% of an individual’s *T*_max_ has been shown to produce significant improvements in aerobic parameters and 3–10 km running performance (Esfarjani and Laursen 2007; Manoel et al. 2017; Smith et al. 2003). In the study by Smith and colleagues (2003), 60% *T*_max_ resulted in an average interval duration of 6 × 133.4 ± 4.1 s. This equated to ~ 13 min of high intensity effort per interval session. In the current study, 7 intervals at 120 s [which also matched the stage duration of the SPV] resulted in ~ 14 min of high intensity effort, ensuring it was comparable to the STND group (See Table 3). Durations of 2 min have been shown to elicit responses closer to $\dot {V}{{\text{O}}_{2\hbox{max} }}$ compared to shorter intervals (O’Brien et al. 2008). Longer interval work periods may have resulted in a greater $\dot {V}{{\text{O}}_{2\hbox{max} }}$ improvement (Esfarjani and Laursen 2007; O’Brien et al. 2008; Seiler and Sjursen 2002) but also significantly increased the interval duration. As a consequence, the mean prescribed training duration for each interval session over the 6 weeks training period was similar between groups (37 ± 8 vs. 38 ± 0 min for STND and S-P, respectively). Total training time over the 6-week period was also similar (804 ± 90 vs. 816 ± 0 min, for STND and S-P, respectively).

The similar $\dot {V}{{\text{O}}_{2\hbox{max} }}$ found between both protocols in this study is in line with previous research (Chidnok et al. 2013; Hogg et al. 2015; Lim et al. 2016; Scheadler and Devor 2015; Straub et al. 2014; Faulkner et al. 2015; Hanson et al. 2016). Even though test duration was significantly longer in the GXT, the test still fell within the recommended duration of 8–12 min (Yoon et al. 2007), and the ${}_{{\text{v}}}\dot {V}{{\text{O}}_{2\hbox{max} }}$ achieved was not significantly different between protocols. Interestingly, RER_max_ was significantly higher in the SPV, which has been observed in some (Mauger and Sculthorpe 2012; Hogg et al. 2015; Jenkins et al. 2017b), but not all previous SPV literature (Lim et al. 2016; Straub et al. 2014; Faulkner et al. 2015; Astorino et al. 2015). Consequently, no consensus on whether the SPV produces a higher RER_max_ can be currently drawn. However, the authors speculate that this potential difference in RER_max_ may be due to the higher peak velocities experienced in the SPV compared to the GXT, indicative of a greater anaerobic contribution towards the end of the test. This is supported by the recent work of Hanson and colleagues (2017) who found, when comparing two SPV trials with different RPE20 pacing strategies, that RER_max_ was significantly greater in the SPV that adopted the more aggressive pacing strategy.

**Table 3:** Training prescription for a representative subject in both training groups

Rep. Subject	Training prescription			
	Interval session × 2		Tempo run	Recovery run
	Weeks 1–3	Weeks 4–6	Weeks 1–6	Weeks 1–6
STND	Work: 6 × 167 s @ 15 km h^− 1^ Recovery: 5 × 334 s @ 8 km h^− 1^	Work: 6 × 141 s @ 16 km h^− 1^ Recovery: 5 × 282 s @ 8 km h^− 1^	30 min @ 11.3 km h^− 1^	30 min @ 115 bpm
S-P	Work: 7 × 120 s @ 15.6 km h^− 1^ Recovery: 6 × 240 s @ 8 km h^− 1^	Work: 7 × 120 s @ 16.3 km h^− 1^ Recovery: 6 × 240 s @ 8 km h^− 1^	30 min @ RPE13	30 min @ 114 bpm

*STND* standardised group, *S-P* self-paced training group

## Conclusions

The ability to prescribe training for recreationally active males and females via SPV-derived parameters offers coaches and athletes valuable alternatives to traditional methods. Prescribing training via the SPV is as effective but more time-economical. Specifically, the same level of improvement in key aerobic fitness parameters can be obtained when training is set via novel training parameters collected from a single 10 min SPV test compared to that achieved using a GXT and a mandatory additional test to acquire *T*_max_ data. This alone may make the SPV more attractive to athletes and coaches, however, recent research regarding a field based SPV (Lim et al. 2016) may emphasise this further. Whilst a field-based SPV has been shown to produce a valid directly measured $\dot {V}{{\text{O}}_{2\hbox{max} }}$, future research should investigate whether $\dot {V}{{\text{O}}_{2\hbox{max} }}$ can be accurately estimated from the field based SPV. If so, athletes and coaches would then be able to utilize a single 10 min test on an athletics track, without expensive equipment, that would offer accurate $\dot {V}{{\text{O}}_{2\hbox{max} }}$ estimation and data for effective training prescription. Therefore, the current findings demonstrate that training parameters derived from the SPV protocol can be used to prescribe effective running training that is similarly effective to training prescribed from GXT-derived parameters. Consequently, in the group that was prescribed training using SPV-derived parameters, $\dot {V}{{\text{O}}_{2\hbox{max} }}$, LTs and CS showed similar improvements compared to runners who were prescribed training via ${}_{{\text{v}}}\dot {V}{{\text{O}}_{2\hbox{max} }}$ and LT zones_,_ with training durations and intensities suitably similar between groups throughout training.

## Abbreviations

ANOVA: Analysis of variance
CS: Critical speed
GPS: Global positioning system
GXT: Graded exercise test
HR_max_: Maximal heart rate
LT: Lactate threshold
LT1: Lactate threshold 1
LT2: Lactate threshold 2
RER: Respiratory exchange ratio
RER_max_: Maximal respiratory exchange ratio
STND: Standardised
S-P: Self-paced
SPV: Self-paced $\dot {V}{{\text{O}}_{{\text{2max}}}}$ test
T_max_: Time in which $_{{\text{v}}}\dot {V}{{\text{O}}_{{\text{2max}}}}$ can be maintained
V_Emax_: Maximal minute ventilation
VCO_2_: Carbon dioxide production
$\dot {V}{{\text{O}}_{{\text{2max}}}}$: Maximal oxygen uptake
$$_{{\text{v}}}\dot {V}{{\text{O}}_{{\text{2max}}}}$$: Velocity at $$\dot {V}{{\text{O}}_{{\text{2max}}}}$$
